# Supplementary material for: High-throughput identification of synthetic riboswitches by barcode-free amplicon-sequencing in human cells
Source: Nat Commun. 2020 Feb 5;11:714. doi: 10.1038/s41467-020-14491-x (PMC7002664; doi:10.1038/s41467-020-14491-x)
Supplement: Supplementary file 1 — Supplementary Information [file 41467_2020_14491_MOESM1_ESM.pdf]

Supplementary Information

High-throughput identification of synthetic riboswitches by barcode-free amplicon-sequencing in human cells

Strobel *et al.*

Supplementary Figure 1

a

cctgcaggcagctgcgcgctcgctcgctcactgaggccgcccgggcaaagcccgggcgtcgggcgacctttggtcgcccggcctcagtgagcgagc
gagcgcgcagagagggagtggccaactccatcactaggggttcctgcggccgcacgcgtctagttattaatagtaatcaattacggggtcattagttcat
agcccatatatggagttccgcgttacataacttacggtaaatggcccgcctggctgaccgcccaacgacccccgcccattgacgtcaataatgacgtatg
ttcccatagtaacgccaatagggactttccattgacgtcaatgggtggagtatttacggtaaactgcccacttggcagtacatcaagtgtatcatatgccaa
gtacgcccccctattgacgtcaatgacggtaaatggcccgcctggcattatgcccagtacatgaccttatgggactttcctacttggcagtacatctacgtatt
agtcatcgctattaccatggtgatgcggttttggcagtacatcaatgggcgtggatagcggtttgactcacggggatttccaagtctccaccccattgacgtc
aatgggagtttgttttgcaccaaaatcaacgggactttccaaaatgtcgtaacaactccgccccattgacgcaaatgggcggtaggcgtgtacggtggga
ggtctatataagcagagctcgtttagtgaaccgtcagatcgcctggagacgccatccacgctgttttgacctccatagaagacaccgggaccgatccag
cctccgcggattcgaacatcgattgaattccccggggatccgccaccatggtgagcaagggcgaggagctgttcaccggggtggtgcccatcctggtc
gagctggacggcgacgtaaacggccacaagttcagcgtgtccggcgagggcgagggcgatgccacctacggcaagctgaccctgaagttcatctgc
accaccggcaagctgcccgtgccctggcccaccctcgtgaccaccctgacctacggcgtgcagtgcttcagccgctaccccgaccacatgaagcagc
acgacttcttcaagtccgccatgcccgaaggctacgtccaggagcgcaccatcttcttcaaggacgacggcaactacaagacccgcgccgaggtgaa
gttcgagggcgacaccctggtgaaccgcatcgagctgaagggcatcgacttcaaggaggacggcaacatcctggggcacaagctggagtacaact
acaacagccacaacgtctatatcatggccgacaagcagaagaacggcatcaaggtgaacttcaagatccgccacaacatcgaggacggcagcgtg
cagctcgccgaccactaccagcagaacacccccatcggcgacggccccgtgctgctgcccgacaaccactacctgagcacccagtccgccctgag
caaagaccccaacgagaagcgcgatcacatggtcctgctggagttcgtgaccgccgccgggatcactctcggcatggacgagctgtacaagtccggc
cggactcagatttcgagctcaagttttgaattttagaagcttggtacccattgcagcgtattcccagtcccaaacaaacaaaggcgcgtcctggattcnnn
nnaaaacataccagatttcgatctggagaggtgaagaatacgaccacctnntacatccagctgatgagtcccaaataggacgaaacgcgctcaaac
aaacaaagcctggtgaaattgttatccgctagatctcttgtttattgcagcttataatggttacaaataaagcaatagcatcacaaatttcacaaataaagca
ttttttcactgcattctagttgtggtttgtccaaactcatcaatgtatcttaacgcggccgcacgtgcggaccgagcggccgcaggaacccctagtgatgga
gttggccactccctctctgcgcgctcgctcgctcactgaggccgggcgaccaaaggtcgcccgacgcccgggctttgcccgggcggcctcagtgagcg
agcgagcgcgcagctgcctgcagg

b

aagcttggtacccattgcagcgtattcccagtcccaaacaaacaaaggccggcatggtcccagcctcctcgctggcgccggctgggcaannntataat
cgcgtggatatggcacgcaagtttctaccgggcaccgtaaatgtccgactannngcgaatgggacgcacaaatctctctagcaaacaaacaaagcct
ggtgaaattgttatccgctagatct

c

aagcttggtacccattgcagcgtattcccagtcccaaacaaacaaaggcgcgtcctggattcnnnnntataatcgcgtggatatggcacgcaagtttcta
ccgggcaccgtaaatgtccgactanntacatccagctgatgagtcccaaataggacgaaacgcgctcaaacaaacaaagcctggtgaaattgttatcc
gctagatct

d

aagcttggtacccattgcagcgtattcccagtcccaaacaaacaaacctttaaagcggttacaagcccgcaaaaatagcagagtaacnnngagaggt
gaagaatacgaccacctaggctcgaaagagcctaaaacatacnnngttaatgcagctttaaaggcaaacaaacaaagcctggtgaaattgttatccg
ctagatct

e

aagcttggtacccattgcagcgtattcccagtcccaaacaaacaaacaggtaagtnnntataatcgcgtggatatggcacgcaagtttctaccgggcac
cgtaaatgtccgactannnacttacctgcaaacaaacaaagcctggtgaaattgttatccgctagatct

AAV2 ITRs | CMV promoter | eGFP | riboswitch | randomized | amplification primer

HindIII and *BglII* RE-sites | inserted in “CG”-design | deleted in “noCG”-design

Fig. 1: Sequences of riboswitch plasmids. (**a**) Tet-hammerhead library plasmid. The same construct with defined instead of randomized nucleotides was also used for the functional characterization of individual aptazyme candidates. (**b**) Gua-HDV library construct. (**c**) Gua-HHR library construct. (**d**) Tet-Twister library construct. The sequence shown here represents the “CG_3N3N” sub-library. Optionally inserted CG pairs in the so-called “CG”- and “noCG”-designs are indicated (see legend). (**e**) Gua-U1 library construct. ITR, inverted terminal repeat, CMV, cytomegalovirus, eGFP, enhanced green fluorescent protein, RE= restriction enzyme.

The data analysed in the context of this study were processed using a computational analysis pipeline implemented in bpipe. The plots and tables in Suppl. Figs. 2-6 and 8 are taken out of the HTML report generated by the pipeline.

Supplementary Figure 2

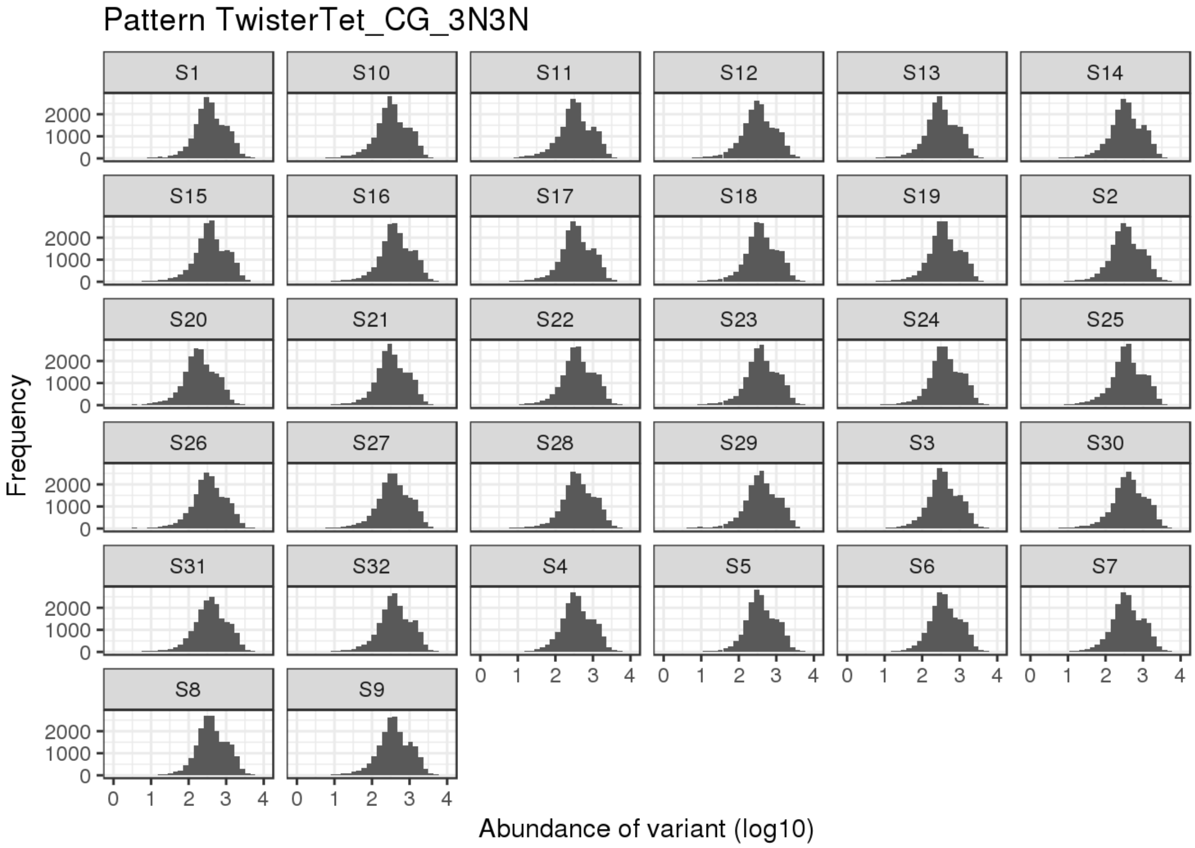

Fig. 2: Per-sample variant abundance of library constructs (quality measure). Exemplarily shown is the analysis for the CG_3N3N Tet-Twister sub-library, screened using 32 replicate samples stimulated with increasing doses of Tet (n=8 per group. S1-S8, untreated; S9-16, 12.5 µM Tet; S17-S24, 25 µM Tet; S25-S32, 50 µM Tet). For each sample, the distribution of raw counts per variant is plotted on a log10 scale. The abundance distribution is consistent across all samples.

Supplementary Figure 3

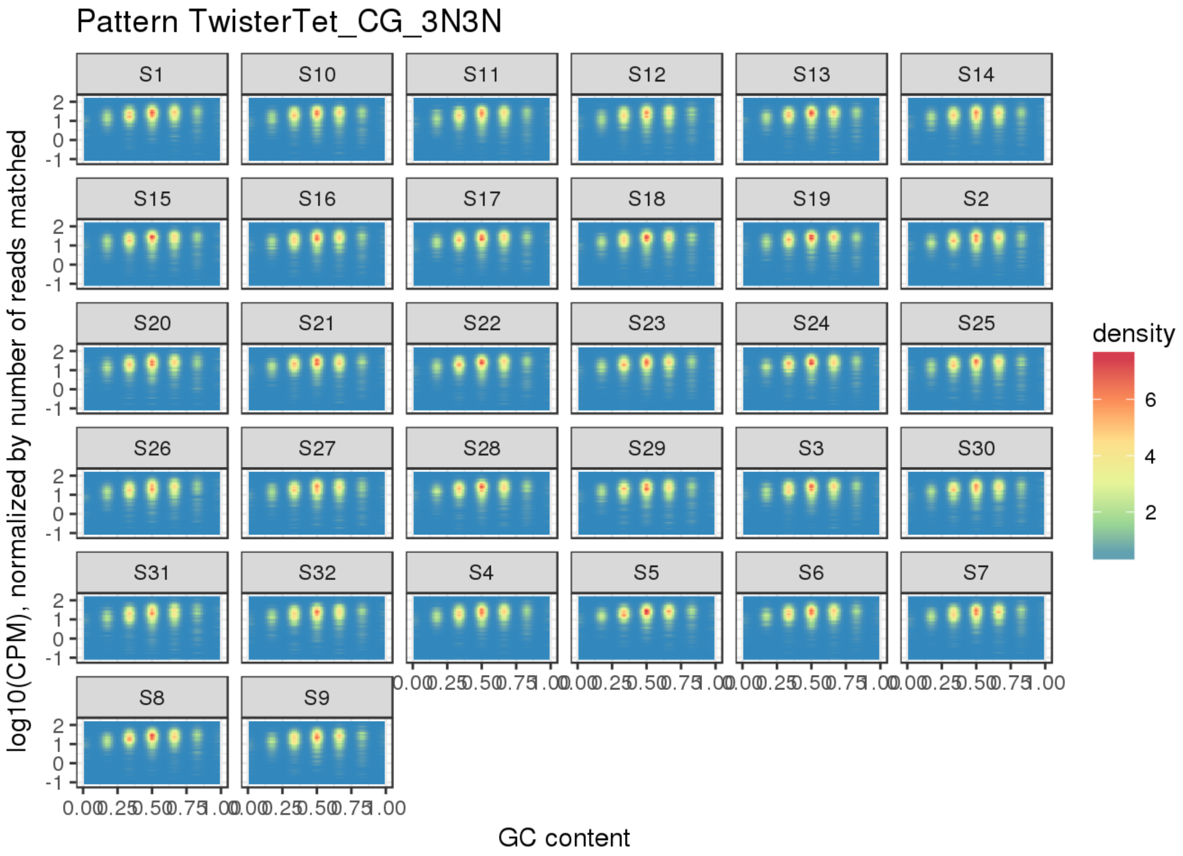

Fig. 3: GC-dependence of variant abundance (quality measure). Per-sample plots of the GC-content per variant vs. the log10(counts per million, CPM) per variant. The discrete values for GC-content result from the limited possibilities for 6 different nucleotides in the CG_3N3N example. The dependence of overall variant abundance on the GC-content is generally low, and the samples do not show differences.

Supplementary Figure 4

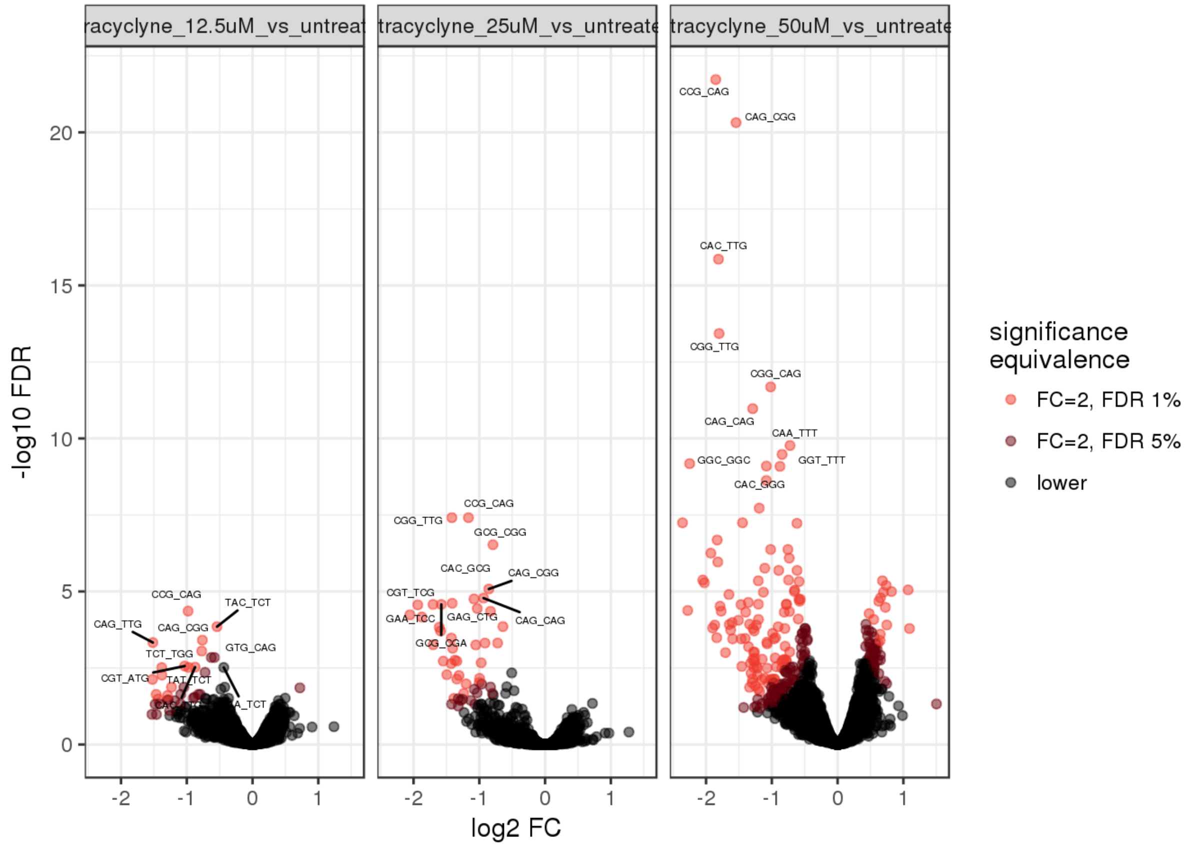

Fig. 4: Volcano plot for differentially abundant variants. Fold-changes and false discovery rate (FDR), as calculated using edgeR for different contrasts (Tet concentrations 12.5 µM, 25 µM and 50 µM vs. untreated). Different significance levels (FDR 1% and 5%) are marked in red. The variable sequences of the variants with the lowest FDRs are annotated in the plot. In the present example of the Tet-Twister sub-library CG_3N3N, the number of variants with differential abundance relative to the untreated control grows with increasing Tet doses.

Supplementary Figure 5

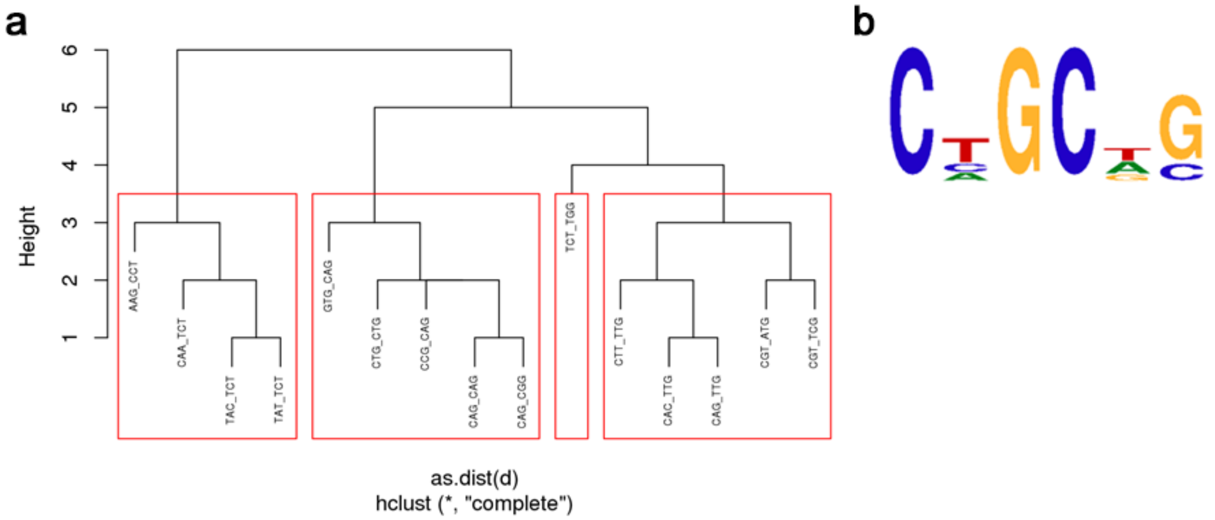

Fig. 5: Sequence similarity analysis. (**a**) Hierarchical clustering of variants with significant differential abundance at an FDR of 1%, based on the Hamming distance. The example shows differentially abundant variants for the Tet-Twister sub-library CG_3N3N for 12.5 µM Tet vs. untreated. (**b**) Position weight matrix of the variable regions of the second cluster.

Supplementary Figure 6

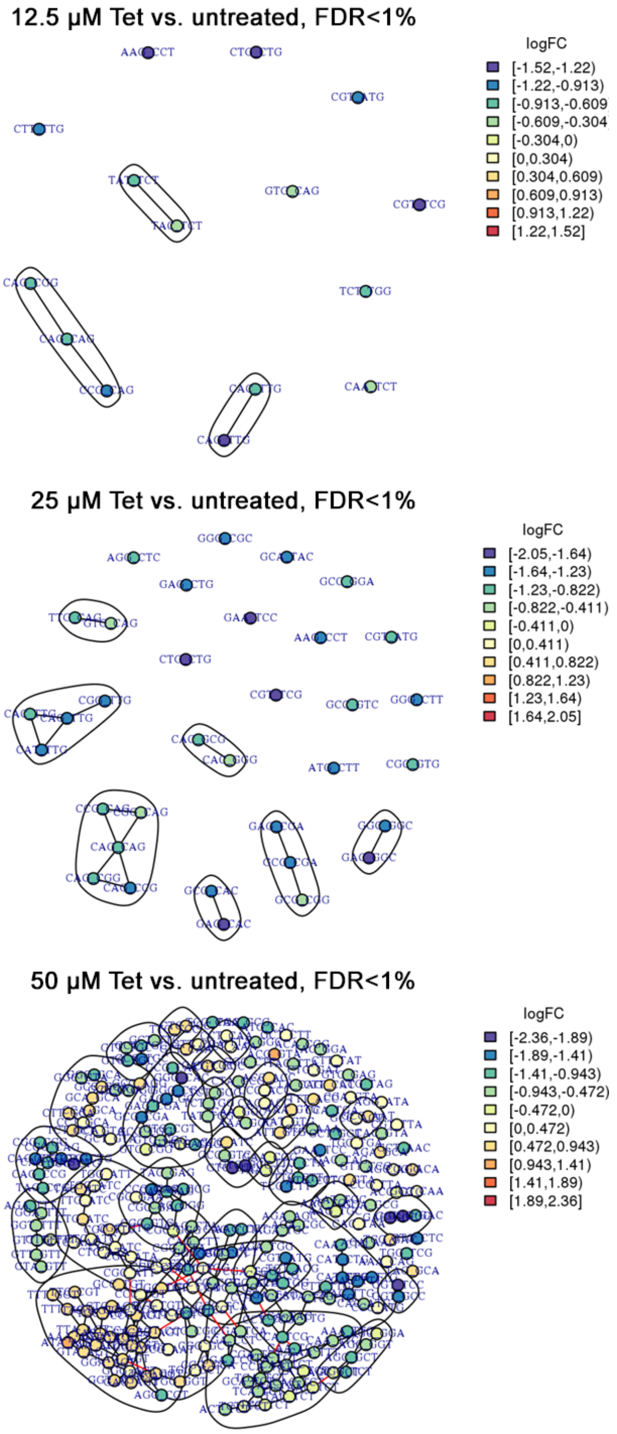

Fig. 6: Network modules of similar variants. Significant differentially abundant motifs are joined if they have a Hamming distance of 1 (single nucleotide difference only). The resulting network is clustered by community inference based on “edge betweenness”. Additionally, the individual variants are colored by logFC. Blue color indicates significantly lower abundance (negative fold change) in the respective contrast, while red color indicates significantly higher abundance (positive fold change). In this example, differentially abundant variants for the Tet-Twister sub-library CG_3N3N at 12.5 µM Tet vs. untreated, 25 µM Tet vs. untreated, and 50 µM Tet vs. untreated at an FDR of 1% are shown.

Supplementary Figure 7

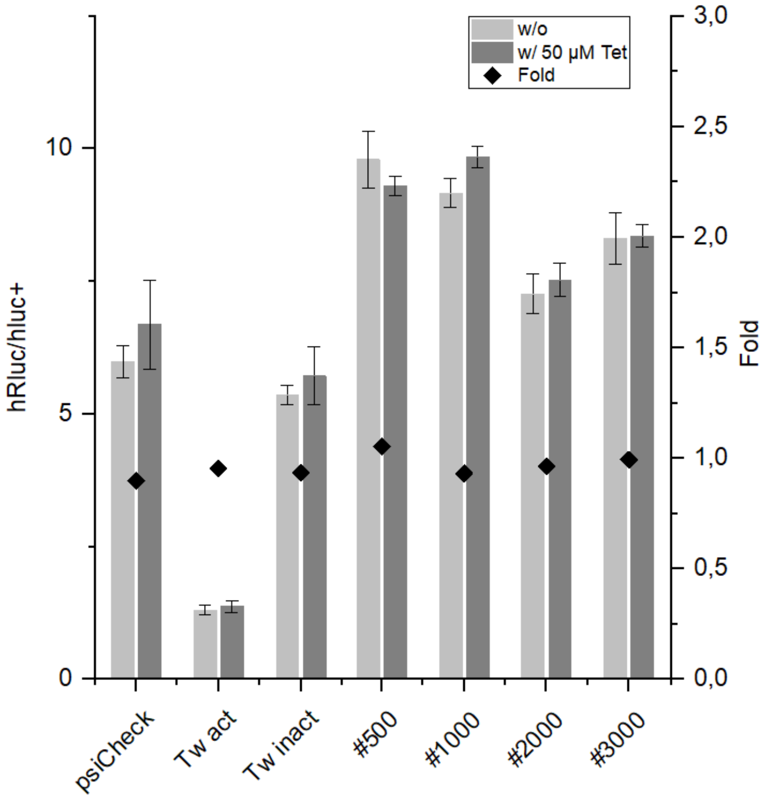

Fig. 7: Switching performance of Twister constructs not fulfilling positive hit selection criteria. Shown are luciferase reporter gene expression data in HeLa cells for the constructs that ranked #500, #1000, #2000 and #3000 in the list of all CG_3N3N constructs that showed a log_2-fold change of >-0.6 and FDR>0.01, sorted by log_2-fold change at 50 µM Tet stimulation (n=3 replicates, mean ± SD). psiCheck: ribozyme-free control; Tw act/inact: constitutively active/inactive ribozyme controls.

Supplementary Figure 8

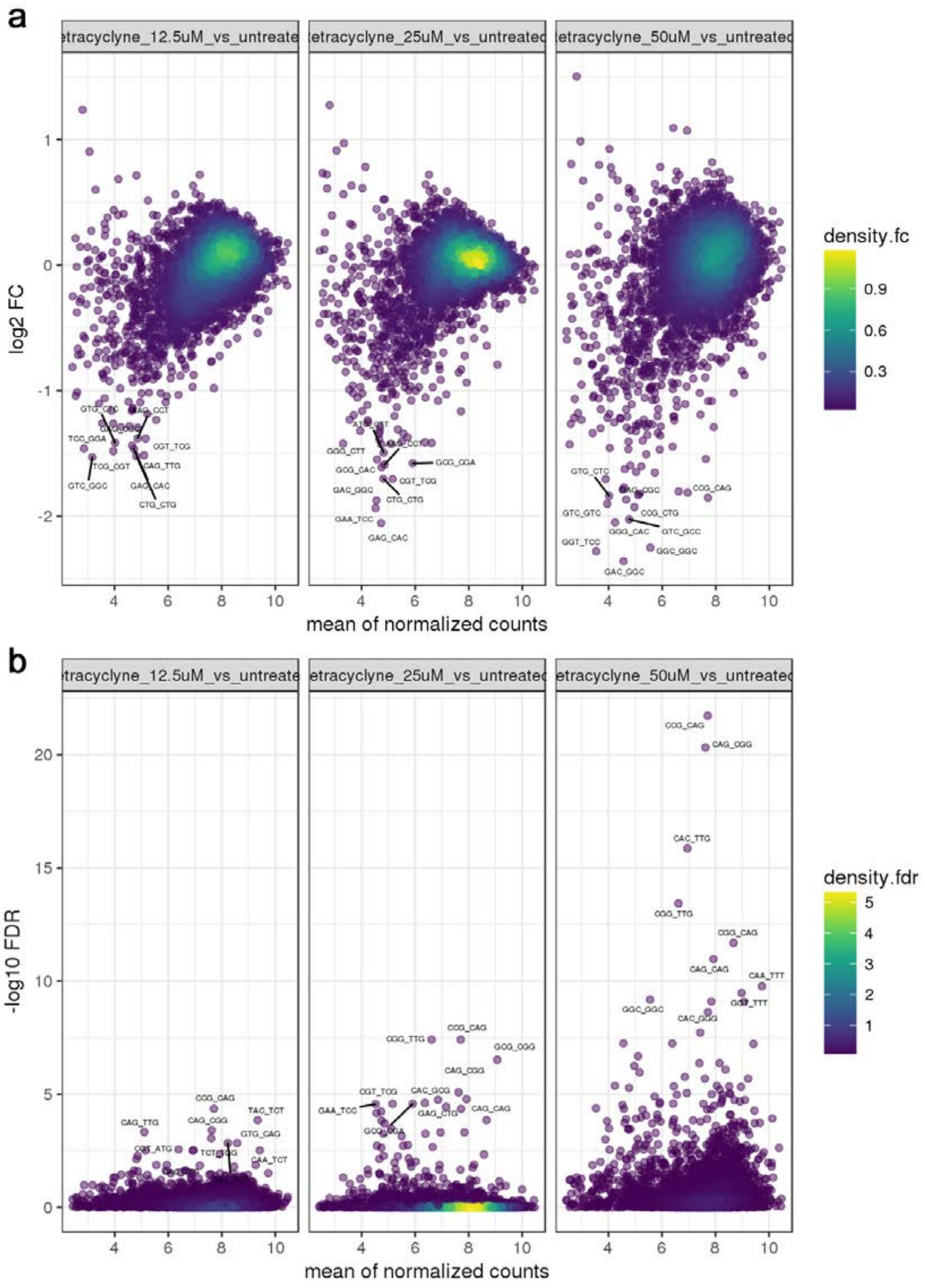

Fig. 8: log_2FC and FDR as a function of construct abundance. Correlation of mean normalized counts with (**a**) log_2FC and (**b**) -log10 FDR, exemplarily shown for the Tet-Twister sub-library CG_3N3N.

Supplementary Figure 9

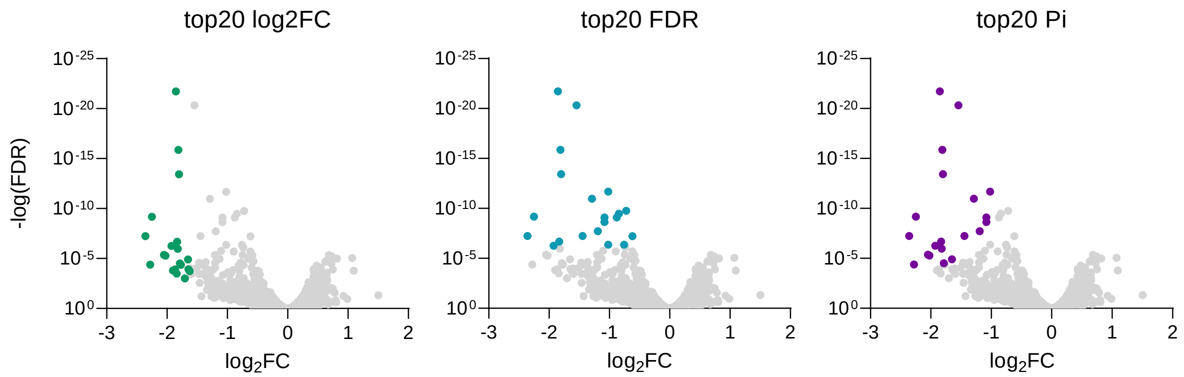

Fig. 9: Dependence of hit selection on selection parameter used. Shown are identical, exemplary volcano plots for the CG_3N3N sub-library contained in the Tet-Twister screen. The top-20 constructs selected based on either ranking of log2FC, FDR or Pi-values are marked in each graph.

Supplementary Figure 10

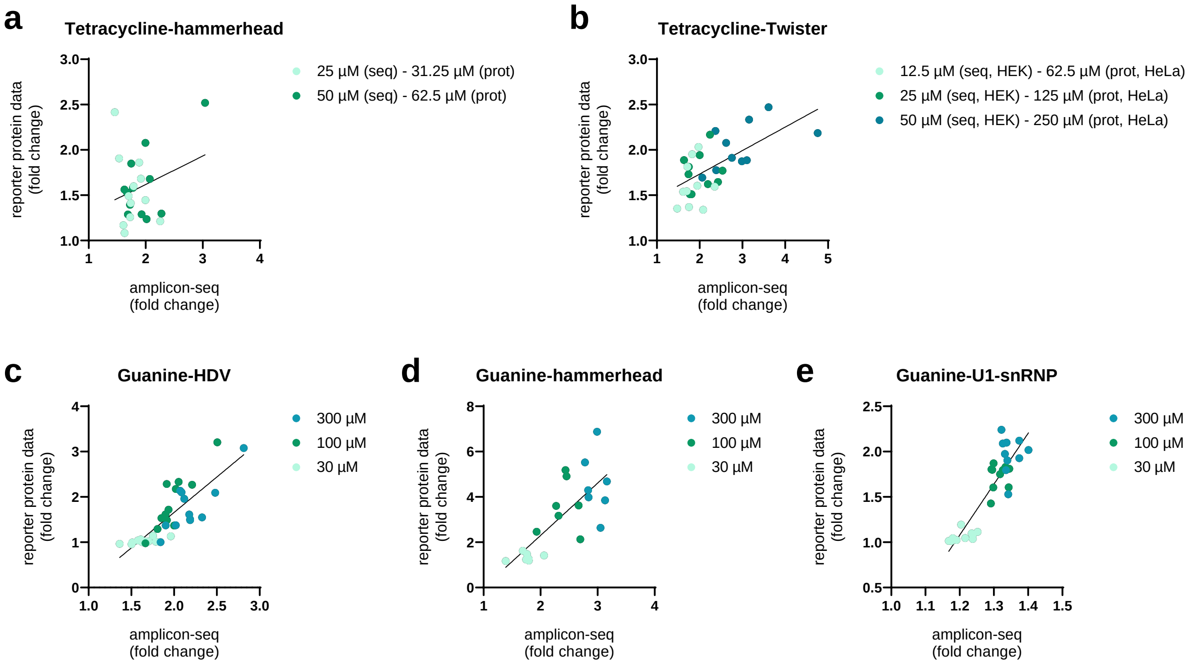

Fig. 10: Correlation of amplicon-seq-derived and experimentally determined (cellular reporter protein assay) mean fold changes for all functional riboswitch constructs. (**a**) Tetracycline hammerhead hit constructs (compare main Fig. 2f). (**b**) Guanine-HDV constructs (compare main Fig. 3d). (**c**) Guanine-hammerhead constructs (compare main Fig. 4d). (**d**) Tetracycline-Twister constructs (compare main Fig. 5c). (**e**) Guanine-U1-snRNP constructs (compare main Fig. 6e). seq= amplicon-seq-derived data, prot= reporter protein assay-derived data. Regression curves were generated using the “linear regression” feature in GraphPad Prism.

Supplementary Table 1

#	Motif	K	logFC 25 µM	logFC 50 µM	logCPM	Pvalue 25 µM	Pvalue 50 µM	FDR 25 µM	FDR 50 µM
1	TAAAC_AG		1,453	2,428	0,944	9,3E-04	3,8E-08	0,330	0,000
2	GTAGT_AC		0,544	1,605	1,961	1,3E-01	9,0E-06	1,000	0,004
3	AATAG_AC		1,031	1,455	2,254	3,5E-03	4,2E-05	0,537	0,012
4	CGTCT_GG		0,756	1,193	2,713	1,8E-02	1,9E-04	0,921	0,040
5	GTATG_CA		1,173	1,185	3,929	4,6E-08	3,4E-08	0,000	0,000
6	GATAA_CC		1,009	1,142	1,800	1,1E-02	3,9E-03	0,777	0,274
7	GTAAA_AC		0,211	1,126	1,288	5,9E-01	3,4E-03	1,000	0,256
8	TAAAT_CT		0,527	1,122	1,919	1,3E-01	1,4E-03	1,000	0,151
9	CGAAC_GG		0,247	1,113	1,950	5,0E-01	2,2E-03	1,000	0,200
10	AAAAA_AT		0,545	1,108	0,998	1,6E-01	4,0E-03	1,000	0,274
11	CATAA_GG		0,841	1,059	1,143	2,4E-02	4,5E-03	0,932	0,288
12	GTGGT_GC		0,937	1,050	5,166	1,8E-11	5,7E-14	0,000	0,000
13	GTAGG_AC		0,938	1,020	2,310	3,8E-03	1,7E-03	0,552	0,170
14	GTAGG_CT		0,703	1,011	5,809	1,5E-08	4,7E-16	0,000	0,000
15	CACCA_CT		0,627	1,002	1,823	6,3E-02	2,9E-03	1,000	0,235
16	CAACC_GG		0,356	1,000	1,411	3,1E-01	4,1E-03	1,000	0,279
17	**GTGGT_AC**	K19	0,617	0,995	3,405	1,8E-02	1,4E-04	0,921	0,031
18	GTGTT_AA		0,394	0,973	4,546	2,5E-02	3,6E-08	0,952	0,000
19	AATAT_AG		0,762	0,953	1,694	3,7E-02	9,1E-03	1,000	0,379
20	GTATT_AA		0,789	0,948	4,680	1,2E-06	6,0E-09	0,003	0,000
21	GTGGC_GT		0,658	0,941	7,750	3,2E-13	2,6E-25	0,000	0,000
22	GTAAG_CT		0,535	0,938	4,849	6,9E-04	3,0E-09	0,277	0,000
23	CAAAT_GG		0,070	0,934	0,375	8,7E-01	3,1E-02	1,000	0,535
24	TAACC_AG		1,310	0,933	1,287	1,1E-03	2,0E-02	0,352	0,467
25	ATAAT_AG		0,411	0,929	1,711	2,5E-01	9,1E-03	1,000	0,379
26	GTGAG_CT		0,660	0,926	5,493	1,5E-07	2,0E-13	0,000	0,000
27	AAAGA_TG		0,561	0,925	2,781	5,6E-02	1,6E-03	1,000	0,168
28	GTAGA_AC		0,452	0,910	2,749	1,3E-01	2,6E-03	1,000	0,223
29	GATAA_CG		0,620	0,909	2,172	7,7E-02	9,6E-03	1,000	0,383
30	TTGAA_CC		0,634	0,905	2,367	5,1E-02	5,5E-03	1,000	0,308
31	CACAT_GG		-0,408	0,884	1,391	3,2E-01	2,8E-02	1,000	0,516
32	CACGA_GG		0,388	0,864	2,372	2,3E-01	7,3E-03	1,000	0,350
33	CAAGG_CC		0,279	0,842	4,139	1,6E-01	1,9E-05	1,000	0,006
34	TAAGA_CG		0,694	0,839	2,790	1,9E-02	4,5E-03	0,921	0,289
35	TAATA_AG		0,883	0,835	0,502	5,8E-02	7,3E-02	1,000	0,649
36	GTACG_CG		0,995	0,827	3,354	5,1E-05	7,6E-04	0,038	0,103
37	TAATA_AT		0,541	0,818	2,323	9,5E-02	1,2E-02	1,000	0,403
38	CTTAA_GG		-0,152	0,814	1,547	6,7E-01	2,0E-02	1,000	0,462
39	GTAGG_TC		0,916	0,804	4,367	6,7E-07	1,3E-05	0,002	0,005
40	GTGAT_AT		0,600	0,803	5,235	1,1E-05	4,3E-09	0,011	0,000
41	CAGTG_GG		-0,290	0,799	1,353	4,5E-01	3,7E-02	1,000	0,555
42	AAACA_TA		-0,453	0,797	1,470	2,1E-01	2,5E-02	1,000	0,506
43	CGATA_AG		0,580	0,797	1,552	1,3E-01	3,7E-02	1,000	0,555
44	TAACG_AG		0,400	0,789	2,048	2,5E-01	2,3E-02	1,000	0,483
45	TCATA_AG		0,618	0,788	1,929	9,0E-02	3,1E-02	1,000	0,535
46	CTAAC_GT		0,797	0,786	3,954	1,0E-04	1,3E-04	0,063	0,029
47	CAGGC_GG		0,203	0,786	0,336	6,0E-01	4,1E-02	1,000	0,574
48	CGAGT_AC		-0,118	0,783	3,480	6,3E-01	1,5E-03	1,000	0,160
49	CGGTA_GG		0,398	0,781	2,675	1,9E-01	9,6E-03	1,000	0,383
50	GTAAC_GA		0,503	0,776	5,690	2,6E-05	9,4E-11	0,021	0,000

#	Motif	K	logFC 25 µM	logFC 50 µM	logCPM	Pvalue 25 µM	Pvalue 50 µM	FDR 25 µM	FDR 50 µM
51	GTAAT_GT		0,484	0,774	5,000	1,8E-03	6,6E-07	0,442	0,000
52	CACTT_CA		0,692	0,772	2,841	1,9E-02	9,1E-03	0,921	0,379
53	CTTTA_CG		0,672	0,771	2,421	4,3E-02	2,0E-02	1,000	0,467
54	TAGTA_CC		0,495	0,769	1,632	1,7E-01	3,2E-02	1,000	0,536
55	ATGAA_CC		0,524	0,767	2,834	9,7E-02	1,5E-02	1,000	0,431
56	AGTTA_AG		0,277	0,760	3,271	3,0E-01	4,8E-03	1,000	0,297
57	CATCC_GG		0,443	0,758	1,230	2,8E-01	6,5E-02	1,000	0,635
58	CTAGT_GC		0,685	0,756	4,858	5,6E-06	5,6E-07	0,008	0,000
59	GTATC_GT		0,336	0,755	5,564	5,8E-03	5,8E-10	0,641	0,000
60	AGACT_AG		0,352	0,752	3,132	1,8E-01	4,5E-03	1,000	0,288
61	GAAGG_AC		0,191	0,752	2,230	5,5E-01	1,8E-02	1,000	0,448
62	CACAA_CT		0,997	0,750	2,293	4,6E-03	3,3E-02	0,599	0,541
63	AGAAC_AA		0,419	0,749	2,080	2,3E-01	3,2E-02	1,000	0,536
64	GTGAA_TT		0,607	0,747	3,806	4,7E-03	5,2E-04	0,611	0,083
65	TGTTA_AA		-0,131	0,747	2,498	6,6E-01	1,2E-02	1,000	0,408
66	**GTATC_GG**	K5	0,527	0,744	5,897	2,7E-05	3,2E-09	0,021	0,000
67	CGCGC_AC		0,599	0,743	1,772	6,3E-02	2,1E-02	1,000	0,475
68	GGAGT_AC		0,767	0,738	4,363	1,5E-05	3,2E-05	0,014	0,010
69	CACTC_GG		0,985	0,734	1,572	1,0E-02	5,6E-02	0,777	0,615
70	TATCA_AG		0,411	0,734	1,914	2,3E-01	3,1E-02	1,000	0,535
71	AGTAT_AA		0,159	0,728	2,181	6,2E-01	2,2E-02	1,000	0,479
72	CGTTA_AG		0,220	0,728	2,515	4,8E-01	1,9E-02	1,000	0,459
73	CAATA_AT		0,344	0,725	2,119	3,1E-01	3,4E-02	1,000	0,543
74	TAGAA_AC		0,337	0,723	0,564	4,0E-01	7,3E-02	1,000	0,649
75	CTATA_CC		-0,398	0,723	1,947	2,3E-01	2,9E-02	1,000	0,526
76	GTACG_CT		0,088	0,720	3,787	6,9E-01	1,3E-03	1,000	0,146
77	CTAGG_CC		0,536	0,715	4,925	6,1E-04	4,9E-06	0,255	0,002
78	CGCAA_GG		0,078	0,713	2,830	7,8E-01	1,1E-02	1,000	0,390
79	CCAAC_AA		-0,088	0,713	1,151	8,2E-01	6,7E-02	1,000	0,636
80	GTGGT_TC		0,605	0,711	4,664	2,2E-04	1,5E-05	0,126	0,005
81	GTGTT_TA		0,403	0,710	3,561	9,0E-02	2,9E-03	1,000	0,233
82	GTAGG_TT		0,422	0,708	5,974	4,5E-04	3,8E-09	0,215	0,000
83	GAGTA_CC		0,366	0,704	2,506	2,5E-01	2,6E-02	1,000	0,509
84	AAAAT_CC		0,532	0,704	2,213	9,4E-02	2,7E-02	1,000	0,514
85	GTGGA_TC		0,837	0,701	5,019	6,3E-08	5,9E-06	0,000	0,002
86	GAAAA_CG		0,590	0,700	0,776	1,5E-01	8,5E-02	1,000	0,677
87	**GTAGT_AT**	K11	0,539	0,699	4,116	9,5E-03	7,8E-04	0,766	0,104
88	GCACC_GG		0,499	0,697	4,703	1,7E-03	1,2E-05	0,441	0,004
89	AAGAG_CC		0,352	0,697	2,025	3,7E-01	7,6E-02	1,000	0,656
90	TAATC_AG		0,987	0,696	2,221	3,8E-03	4,1E-02	0,552	0,574
91	AAAAT_AG		-0,265	0,695	0,833	5,6E-01	1,3E-01	1,000	0,724
92	GATAT_CT		0,511	0,694	3,358	3,5E-02	4,3E-03	0,998	0,287
93	CTATT_AA		0,178	0,690	3,261	5,2E-01	1,3E-02	1,000	0,409
94	GGTAA_CC		0,077	0,690	2,316	8,1E-01	3,4E-02	1,000	0,543
95	CACTT_GG		-0,498	0,689	1,771	1,6E-01	4,8E-02	1,000	0,606
96	CAGCC_GG		0,276	0,688	3,694	2,2E-01	2,2E-03	1,000	0,200
97	TAGTC_AG		1,218	0,685	1,578	6,2E-04	5,4E-02	0,255	0,614
98	GACGC_GC		0,396	0,685	1,870	2,2E-01	3,3E-02	1,000	0,541
99	GTACG_AC		0,731	0,685	2,510	1,8E-02	2,7E-02	0,921	0,514
100	CGATT_CT		0,528	0,684	3,019	5,1E-02	1,2E-02	1,000	0,403

Tab. 1: Top 100 constructs from the Tet-hammerhead screen, ranked by their fold change at 50 µM Tet (here, no further cutoffs were applied). K* constructs were contained in the original publication by Beilstein and colleagues.

Supplementary Table 2

#	Motif	GuaM*	logCPM	logFC 30 µM	logFC 100 µM	logFC 300 µM	P-value 30 µM	P-value 100 µM	P-value 300 µM	FDR 30 µM	FDR 100 µM	FDR 300 µM
1	TGC_GTA	GuaM8HDV	6.68	-0.971	-1.325	-1.492	2.20E-16	2.20E-16	2.20E-16	2.05E-14	1.50E-14	1.07E-14
2	TGG_CTA		6.68	-0.837	-1.144	-1.311	2.20E-16	2.20E-16	2.20E-16	2.05E-14	1.50E-14	1.07E-14
3	TGA_TTA		8.16	-0.588	-1.000	-1.218	2.20E-16	2.20E-16	2.20E-16	2.05E-14	1.50E-14	1.07E-14
4	CGG_CTG		5.19	-0.768	-0.926	-1.129	2.20E-16	2.20E-16	2.20E-16	2.05E-14	1.50E-14	1.07E-14
5	CGG_TCG		5.39	-0.703	-0.951	-1.129	2.20E-16	2.20E-16	2.20E-16	2.05E-14	1.50E-14	1.07E-14
6	TGT_ATA	GuaM12HDV	7.58	-0.447	-0.852	-1.121	2.20E-16	2.20E-16	2.20E-16	2.05E-14	1.50E-14	1.07E-14
7	CGT_ACG		5.25	-0.687	-0.937	-1.083	2.20E-16	2.20E-16	2.20E-16	2.05E-14	1.50E-14	1.07E-14
8	CGC_GCG		3.99	-0.809	-1.038	-1.061	2.20E-16	2.20E-16	2.20E-16	2.05E-14	1.50E-14	1.07E-14
9	CGT_GCG		3.83	-0.728	-1.014	-1.050	2.20E-16	2.20E-16	2.20E-16	2.05E-14	1.50E-14	1.07E-14
10	CAA_TTG		7.01	-0.656	-0.888	-1.014	2.20E-16	2.20E-16	2.20E-16	2.05E-14	1.50E-14	1.07E-14
11	CGC_GTG		4.18	-0.596	-0.938	-0.927	2.20E-16	2.20E-16	2.20E-16	2.05E-14	1.50E-14	1.07E-14
12	CAC_GTG		5.50	-0.594	-0.736	-0.881	2.20E-16	2.20E-16	2.20E-16	2.05E-14	1.50E-14	1.07E-14
13	CGA_TCG		5.27	-0.663	-0.777	-0.880	2.20E-16	2.20E-16	2.20E-16	2.05E-14	1.50E-14	1.07E-14
14	CGA_TTG		6.00	-0.423	-0.673	-0.878	2.20E-16	2.20E-16	2.20E-16	2.05E-14	1.50E-14	1.07E-14
15	CGA_CCG		6.76	-0.303	-0.678	-0.877	2.20E-16	2.20E-16	2.20E-16	2.05E-14	1.50E-14	1.07E-14
16	CGT_ATG		6.56	-0.405	-0.642	-0.828	2.20E-16	2.20E-16	2.20E-16	2.05E-14	1.50E-14	1.07E-14
17	CGG_CCG		4.35	-0.591	-0.845	-0.800	2.20E-16	2.20E-16	2.20E-16	2.05E-14	1.50E-14	1.07E-14
18	CGC_ACG		6.06	-0.221	-0.540	-0.798	1.22E-08	2.20E-16	2.20E-16	9.27E-07	1.50E-14	1.07E-14
19	CCA_TGG		4.14	-0.527	-0.648	-0.780	2.20E-16	2.20E-16	2.20E-16	2.05E-14	1.50E-14	1.07E-14
20	CAT_ATG		5.97	-0.495	-0.614	-0.756	2.20E-16	2.20E-16	2.20E-16	2.05E-14	1.50E-14	1.07E-14
21	CAG_CTG		5.60	-0.494	-0.612	-0.739	2.20E-16	2.20E-16	2.20E-16	2.05E-14	1.50E-14	1.07E-14
22	TGG_TTA		8.13	-0.243	-0.480	-0.733	2.20E-16	2.20E-16	2.20E-16	2.05E-14	1.50E-14	1.07E-14
23	CCG_TGG		3.92	-0.440	-0.534	-0.732	1.08E-10	7.56E-15	2.20E-16	8.81E-09	4.92E-13	1.07E-14
24	CGA_GCG		6.22	-0.095	-0.436	-0.718	1.01E-02	2.20E-16	2.20E-16	2.36E-01	1.50E-14	1.07E-14
25	CAG_TTG		6.39	-0.283	-0.519	-0.696	2.20E-16	2.20E-16	2.20E-16	2.05E-14	1.50E-14	1.07E-14
26	TGT_ACA		8.08	-0.430	-0.555	-0.682	2.20E-16	2.20E-16	2.20E-16	2.05E-14	1.50E-14	1.07E-14
27	CAT_GTG		5.76	-0.317	-0.494	-0.680	2.20E-16	2.20E-16	2.20E-16	2.05E-14	1.50E-14	1.07E-14
28	CGG_GCG		4.83	-0.144	-0.387	-0.675	4.08E-03	3.47E-14	2.20E-16	1.38E-01	2.18E-12	1.07E-14
29	CGT_CCG		5.63	-0.218	-0.429	-0.664	1.05E-07	2.20E-16	2.20E-16	7.65E-06	1.50E-14	1.07E-14
30	CTA_TAG		7.42	-0.431	-0.563	-0.636	2.20E-16	2.20E-16	2.20E-16	2.05E-14	1.50E-14	1.07E-14
31	TGT_GCA		7.39	-0.319	-0.501	-0.636	2.20E-16	2.20E-16	2.20E-16	2.05E-14	1.50E-14	1.07E-14
32	CGG_TTG		6.57	-0.163	-0.378	-0.601	1.38E-09	2.20E-16	2.20E-16	1.09E-07	1.50E-14	1.07E-14
33	CGT_TCG		6.15	-0.163	-0.449	-0.599	5.22E-08	2.20E-16	2.20E-16	3.89E-06	1.50E-14	1.07E-14
34	TGA_TCA		8.43	-0.425	-0.561	-0.597	2.20E-16	2.20E-16	2.20E-16	2.05E-14	1.50E-14	1.07E-14
35	CAG_CCG		6.57	-0.242	-0.509	-0.595	2.20E-16	2.20E-16	2.20E-16	2.05E-14	1.50E-14	1.07E-14
36	TGC_GCA		6.83	-0.461	-0.591	-0.579	2.20E-16	2.20E-16	2.20E-16	2.05E-14	1.50E-14	1.07E-14
37	CGG_ACG		6.36	-0.117	-0.357	-0.560	2.92E-05	2.20E-16	2.20E-16	1.96E-03	1.50E-14	1.07E-14
38	TCA_TGA		7.11	-0.391	-0.523	-0.549	2.20E-16	2.20E-16	2.20E-16	2.05E-14	1.50E-14	1.07E-14
39	TCG_TGA		6.22	-0.286	-0.418	-0.536	2.20E-16	2.20E-16	2.20E-16	2.05E-14	1.50E-14	1.07E-14
40	CTC_GAG		5.91	-0.317	-0.449	-0.502	2.20E-16	2.20E-16	2.20E-16	2.05E-14	1.50E-14	1.07E-14
41	CCT_AGG		5.93	-0.382	-0.448	-0.492	2.20E-16	2.20E-16	2.20E-16	2.05E-14	1.50E-14	1.07E-14
42	CGA_ACG		6.24	-0.091	-0.190	-0.470	1.21E-02	1.82E-07	2.20E-16	2.51E-01	8.98E-06	1.07E-14
43	CAC_GCG		4.53	-0.320	-0.397	-0.466	8.29E-09	1.33E-12	2.20E-16	6.41E-07	8.00E-11	1.07E-14
44	CCG_CGG		3.35	-0.182	-0.310	-0.460	1.99E-02	8.69E-05	7.02E-09	3.36E-01	2.95E-03	2.55E-07
45	CCT_GGG		5.14	-0.167	-0.412	-0.456	1.41E-03	7.93E-15	2.20E-16	6.08E-02	5.08E-13	1.07E-14
46	TCC_GGA		6.52	-0.299	-0.392	-0.447	2.20E-16	2.20E-16	2.20E-16	2.05E-14	1.50E-14	1.07E-14
47	TGG_TCA		7.66	-0.259	-0.362	-0.444	2.20E-16	2.20E-16	2.20E-16	2.05E-14	1.50E-14	1.07E-14
48	CTT_AAG		7.76	-0.258	-0.363	-0.443	2.20E-16	2.20E-16	2.20E-16	2.05E-14	1.50E-14	1.07E-14
49	CGC_TCG		4.83	-0.143	-0.263	-0.437	7.64E-03	1.09E-06	9.71E-16	2.09E-01	4.85E-05	4.57E-14
50	TCT_AGA		7.55	-0.273	-0.374	-0.422	2.20E-16	2.20E-16	2.20E-16	2.05E-14	1.50E-14	1.07E-14
51	TTC_GTA		7.83	-0.174	-0.319	-0.418	2.20E-16	2.20E-16	2.20E-16	2.05E-14	1.50E-14	1.07E-14
52	TAA_TTA	GuaM7HDV	8.82	-0.186	-0.323	-0.394	2.20E-16	2.20E-16	2.20E-16	2.05E-14	1.50E-14	1.07E-14
53	CCC_GGG		5.65	-0.305	-0.435	-0.394	2.20E-16	2.20E-16	2.20E-16	2.05E-14	1.50E-14	1.07E-14
54	CCT_TGG		6.37	-0.074	-0.279	-0.385	2.11E-02	2.20E-16	2.20E-16	3.40E-01	1.50E-14	1.07E-14
55	TCG_CGA		6.31	-0.275	-0.394	-0.371	4.27E-12	2.20E-16	2.20E-16	3.64E-10	1.50E-14	1.07E-14
56	CGC_CCG		4.58	-0.157	-0.260	-0.364	1.99E-02	1.21E-04	8.24E-08	3.36E-01	3.87E-03	2.84E-06
57	CCA_CGG		5.34	-0.067	-0.161	-0.356	1.65E-01	9.35E-04	4.04E-13	7.32E-01	2.29E-02	1.78E-11
58	TCT_GGA		7.51	-0.087	-0.208	-0.342	1.29E-04	2.20E-16	2.20E-16	8.01E-03	1.50E-14	1.07E-14
59	TGT_GTA	GuaM11HDV	8.05	-0.059	-0.185	-0.341	9.72E-04	2.20E-16	2.20E-16	4.37E-02	1.50E-14	1.07E-14
60	TAC_GTA	GuaM1HDV	7.24	-0.240	-0.286	-0.336	2.20E-16	2.20E-16	2.20E-16	2.05E-14	1.50E-14	1.07E-14

Tab. 2: Top 60 constructs from the Gua-HDV screen, ranked by their fold change at 300 µM Gua (here, no further cutoffs were applied). GuaM* constructs were contained in the original publication by Nomura and colleagues.

Supplementary Table 3

Sub-Library	#	Motif	logCPM	log2FC 12.5 µM	log2FC 25 µM	log2FC 50 µM	p value 12.5 µM	p value 25 µM	p value 50 µM	FDR 12.5 µM	FDR 25 µM	FDR 50 µM
2N3N	1	CG_CGG	7,718	-0,808	-0,856	-1,043	1,15E-10	7,88E-12	7,20E-17	5,86E-08	8,07E-09	3,69E-14
	2	GG_CCA	7,933	-0,429	-0,532	-0,916	7,02E-04	2,65E-05	5,81E-13	1,24E-02	5,42E-03	7,44E-11
	3	GG_CCG	7,800	-0,553	-0,580	-0,908	5,05E-07	1,27E-07	1,56E-16	3,77E-05	4,34E-05	3,99E-14
	4	CG_CGC	7,666	-0,650	-0,451	-0,804	6,46E-08	1,50E-04	1,60E-11	8,06E-06	2,20E-02	1,64E-09
	5	CG_CGA	7,299	-0,832	-0,771	-0,755	1,99E-10	2,98E-09	4,19E-09	6,81E-08	1,53E-06	2,68E-07
	6	CC_GGT	7,966	-0,528	-0,243	-0,697	1,57E-06	2,55E-02	1,83E-10	8,92E-05	4,95E-01	1,25E-08
	7	CG_CGT	6,538	-0,614	-0,526	-0,656	3,57E-04	2,05E-03	1,11E-04	7,46E-03	1,34E-01	1,23E-03
	8	GC_GCG	9,099	-0,361	-0,342	-0,624	3,19E-06	9,65E-06	6,33E-16	1,63E-04	2,47E-03	1,30E-13
	9	GA_TCG	8,641	-0,480	-0,276	-0,620	6,61E-07	4,02E-03	1,09E-10	3,98E-05	1,96E-01	8,61E-09
	10	TC_CTG	9,973	-0,103	-0,017	-0,580	1,83E-01	8,28E-01	9,21E-14	4,34E-01	9,68E-01	1,35E-11
3N2N	1	ACG_CG	6,553	-1,380	-1,178	-1,545	6,15E-24	1,54E-18	2,51E-30	6,30E-21	5,27E-16	2,57E-27
	2	CGG_CC	7,354	-0,763	-0,804	-1,389	6,43E-08	1,13E-08	2,52E-22	9,40E-06	1,65E-06	6,45E-20
	3	AAG_CT	7,061	-1,043	-1,139	-1,297	9,00E-16	1,49E-18	9,78E-24	4,61E-13	5,27E-16	3,34E-21
	4	GCG_CG	7,879	-0,698	-0,747	-1,178	6,25E-10	3,32E-11	3,23E-25	1,72E-07	6,81E-09	1,65E-22
	5	ACC_GG	7,172	-0,790	-0,648	-0,979	1,61E-09	5,91E-07	5,10E-14	3,31E-07	4,32E-05	5,23E-12
	6	GTG_CC	7,381	-0,534	-0,700	-0,973	5,17E-05	1,16E-07	1,85E-13	1,41E-03	1,19E-05	1,72E-11
	7	GCC_GG	7,462	-0,779	-0,822	-0,939	1,44E-08	1,96E-09	6,38E-12	2,46E-06	3,35E-07	5,03E-10
	8	AGC_GC	7,215	-0,621	-0,860	-0,921	2,58E-07	1,19E-12	1,74E-14	2,20E-05	3,05E-10	1,98E-12
	9	CCG_CG	9,043	-0,299	-0,295	-0,902	2,63E-03	2,89E-03	1,85E-19	3,02E-02	7,97E-02	3,80E-17
	10	GGC_GC	7,465	-0,637	-0,538	-0,879	1,54E-05	2,37E-04	2,18E-09	5,43E-04	1,01E-02	9,69E-08
3N3N	1	GCG_CGT	4,243	-0,999	-1,208	-2,150	1,35E-04	4,17E-06	5,19E-15	1,88E-02	5,18E-03	2,13E-11
	2	GTG_TAC	4,744	-0,665	-0,658	-2,017	1,26E-02	1,28E-02	6,51E-13	1,96E-01	4,91E-01	6,67E-10
	3	GAG_CTT	4,885	-0,960	-1,132	-1,579	3,06E-05	9,64E-07	1,35E-11	8,97E-03	2,88E-03	9,21E-09
	4	ACG_CGG	5,579	-1,037	-1,023	-1,557	3,18E-05	3,71E-05	6,32E-10	8,97E-03	1,90E-02	2,27E-07
	5	GGG_CTC	3,384	-0,784	-0,322	-1,544	1,48E-02	2,95E-01	2,36E-06	2,04E-01	9,26E-01	1,90E-04
	6	CCG_TGG	5,987	-0,723	-0,602	-1,369	1,35E-04	1,32E-03	1,03E-12	1,88E-02	1,29E-01	8,47E-10
	7	TGG_CTA	4,911	-1,002	-0,767	-1,343	4,09E-05	1,38E-03	3,61E-08	8,97E-03	1,29E-01	5,48E-06
	8	CGA_TTG	5,561	-0,593	-0,469	-1,292	4,31E-03	2,26E-02	8,95E-10	1,14E-01	5,54E-01	2,63E-07
	9	TGC_GTA	5,699	-0,341	-0,421	-1,275	1,21E-01	5,50E-02	1,49E-08	5,51E-01	7,26E-01	2,77E-06
	10	CTG_CAG	4,401	-0,858	-0,788	-1,271	5,22E-04	1,25E-03	2,91E-07	3,69E-02	1,25E-01	3,41E-05
CG_2N3N	1	CG_CGA	7,013	-0,868	-1,000	-1,658	2,13E-06	4,41E-08	6,76E-19	2,96E-04	9,04E-06	2,31E-16
	2	CG_CGG	8,119	-1,059	-1,279	-1,634	4,22E-14	9,29E-20	1,07E-30	4,32E-11	9,51E-17	1,09E-27
	3	CG_CTG	6,517	-0,416	0,017	-0,993	4,46E-02	9,34E-01	2,06E-06	2,14E-01	9,96E-01	6,20E-05
	4	CG_CGC	7,709	-0,641	-0,568	-0,965	7,93E-06	6,52E-05	1,65E-11	5,07E-04	5,13E-03	1,87E-09
	5	CA_TGG	8,928	-0,693	-0,618	-0,887	3,56E-10	1,83E-08	7,83E-16	1,22E-07	4,68E-06	1,60E-13
	6	GA_TCA	7,259	-0,381	-0,362	-0,811	2,21E-02	2,87E-02	1,16E-06	1,37E-01	4,41E-01	3,83E-05
	7	CG_CGT	7,647	-0,761	-0,526	-0,772	4,25E-06	1,30E-03	2,38E-06	3,35E-04	5,12E-02	6,95E-05
	8	GG_CTG	7,987	-0,522	-0,562	-0,758	1,02E-03	3,92E-04	1,70E-06	1,96E-02	2,36E-02	5,43E-05
	9	TG_CAG	10,113	-0,352	-0,543	-0,749	2,03E-06	2,48E-13	5,86E-24	2,96E-04	1,27E-10	3,00E-21
	10	AG_TGC	10,827	0,006	0,042	-0,708	9,45E-01	6,19E-01	1,76E-16	9,81E-01	9,62E-01	4,50E-14
CG_3N2N	1	GAG_CC	5,244	-0,653	-0,804	-1,593	1,51E-03	8,61E-05	6,56E-14	1,60E-02	3,27E-03	5,17E-12
	2	CCG_CG	7,089	-0,688	-0,799	-1,462	1,95E-06	2,95E-08	3,03E-23	8,70E-05	4,32E-06	7,75E-21
	3	CGG_CC	6,660	-0,812	-0,741	-1,358	6,40E-07	4,23E-06	1,35E-16	3,45E-05	2,40E-04	1,97E-14
	4	GCG_CC	6,370	-1,157	-1,468	-1,245	4,45E-10	3,87E-15	8,83E-12	5,70E-08	2,24E-12	5,65E-10
	5	CAG_CT	8,381	-0,774	-0,709	-1,244	5,79E-12	2,07E-10	2,99E-28	1,97E-09	5,30E-08	1,53E-25
	6	CGT_CG	8,188	-0,500	-0,652	-1,226	1,80E-05	2,17E-08	3,34E-25	6,83E-04	3,71E-06	1,14E-22
	7	CGA_TC	8,501	-0,572	-0,555	-0,963	1,71E-07	3,31E-07	1,33E-18	1,35E-05	2,83E-05	2,72E-16
	8	ACG_CG	7,152	-0,779	-0,580	-0,954	7,54E-08	4,67E-05	2,87E-11	7,02E-06	1,91E-03	1,55E-09
	9	GAA_TC	5,711	-0,590	-0,619	-0,945	1,99E-03	1,03E-03	6,29E-07	1,88E-02	2,93E-02	1,43E-05
	10	CTG_CA	8,284	-0,460	-0,516	-0,940	6,96E-05	7,43E-06	5,51E-16	1,84E-03	3,62E-04	5,64E-14
CG_3N3N	1	GAC_GGC	4,558	-1,282	-1,874	-2,358	2,98E-04	2,34E-07	2,02E-10	3,49E-02	6,85E-05	5,69E-08
	2	GGT_TCC	3,535	-1,260	-0,577	-2,279	3,65E-03	1,61E-01	5,30E-07	1,13E-01	7,66E-01	4,17E-05
	3	GGC_GGC	5,555	-1,233	-1,342	-2,250	5,87E-05	1,16E-05	1,47E-12	1,34E-02	1,89E-03	6,67E-10
	4	GGG_CAC	4,242	-1,291	-1,254	-2,049	3,21E-04	3,90E-04	2,80E-08	3,55E-02	3,33E-02	4,24E-06
	5	GTC_GCC	4,769	-0,673	-0,657	-2,025	5,48E-02	5,84E-02	3,94E-08	3,49E-01	5,64E-01	5,21E-06
	6	CCG_CTG	4,961	-1,093	-1,067	-1,927	5,66E-04	6,67E-04	2,74E-09	4,73E-02	4,80E-02	5,61E-07
	7	GTC_GTC	3,949	-1,261	-1,318	-1,900	1,54E-03	8,32E-04	2,67E-06	7,83E-02	5,59E-02	1,56E-04
	8	GAG_CGC	4,656	-1,436	-1,386	-1,868	2,48E-04	3,51E-04	2,05E-06	3,28E-02	3,13E-02	1,25E-04
	9	CCG_CAG	7,707	-0,980	-1,165	-1,852	1,06E-08	1,16E-11	4,61E-26	4,36E-05	3,88E-08	1,89E-22
	10	GTG_CTC	4,020	-1,415	-1,162	-1,838	5,63E-04	3,55E-03	7,14E-06	4,73E-02	1,45E-01	3,25E-04
noCG_2N3N	1	CT_GCG	9,823	-0,216	-0,046	-0,730	3,18E-03	5,27E-01	2,85E-23	5,93E-02	9,88E-01	1,46E-20
	2	CT_AGC	10,795	-0,127	-0,084	-0,686	3,88E-02	1,71E-01	8,11E-29	2,78E-01	9,72E-01	8,31E-26
	3	CT_GGT	10,832	-0,200	-0,109	-0,634	1,77E-02	1,97E-01	6,11E-14	1,93E-01	9,77E-01	6,25E-12
	4	TC_GGT	9,970	-0,455	-0,087	-0,614	3,22E-13	1,57E-01	4,85E-23	1,65E-10	9,59E-01	1,65E-20
	5	CC_GGT	9,476	-0,700	-0,363	-0,614	2,31E-15	3,29E-05	2,28E-12	2,37E-12	2,06E-02	1,55E-10
	6	CA_TGC	10,526	-0,034	0,027	-0,596	6,67E-01	7,39E-01	1,06E-13	8,60E-01	9,88E-01	9,89E-12
	7	CT_TGT	9,719	-0,105	-0,158	-0,564	2,24E-01	6,70E-02	6,69E-11	5,71E-01	9,06E-01	3,26E-09
	8	CA_TGT	10,082	-0,137	-0,101	-0,562	1,24E-01	2,57E-01	3,63E-10	4,57E-01	9,88E-01	1,49E-08
	9	AC_AGC	10,895	-0,246	-0,089	-0,556	7,21E-05	1,51E-01	2,83E-19	4,10E-03	9,59E-01	7,25E-17
	10	AC_GGC	11,689	-0,523	-0,130	-0,547	6,77E-13	7,35E-02	5,05E-14	2,31E-10	9,16E-01	5,75E-12
noCG_3N2N	1	AGG_CC	7,795	-0,647	-0,729	-0,968	1,97E-08	2,29E-10	3,31E-17	1,91E-05	2,35E-07	3,38E-14
	2	AGC_GC	8,031	-0,543	-0,467	-0,649	6,14E-07	1,59E-05	1,70E-09	9,09E-05	8,14E-03	1,25E-07
	3	CGC_GC	9,553	-0,268	-0,158	-0,621	1,46E-03	6,05E-02	1,64E-13	3,65E-02	6,88E-01	8,38E-11
	4	AGA_TC	8,033	-0,431	-0,451	-0,529	6,80E-05	2,91E-05	8,01E-07	3,48E-03	9,95E-03	2,83E-05
	5	ATC_GG	10,023	-0,371	-0,217	-0,528	8,16E-07	3,74E-03	1,60E-12	1,05E-04	2,74E-01	3,32E-10
	6	ACC_GG	9,809	-0,464	-0,330	-0,519	3,73E-08	8,40E-05	5,90E-10	1,91E-05	2,15E-02	5,49E-08
	7	TCA_GG	10,522	-0,325	-0,064	-0,505	6,01E-06	3,70E-01	1,62E-12	5,46E-04	9,60E-01	3,32E-10
	8	CCT_AG	9,374	-0,195	-0,152	-0,498	1,90E-02	6,78E-02	2,17E-09	2,02E-01	6,88E-01	1,48E-07
	9	GGT_CC	4,997	-0,266	0,250	-0,493	1,58E-01	1,64E-01	7,21E-03	5,55E-01	9,30E-01	4,47E-02
	10	AGA_GC	10,135	-0,102	-0,067	-0,480	1,27E-01	3,13E-01	7,42E-13	4,95E-01	9,60E-01	2,53E-10
noCG_3N3N	1	GGG_TCC	5,214	-0,676	-1,121	-1,673	1,78E-03	3,35E-07	6,09E-14	1,46E-01	3,43E-04	6,24E-11
	2	GGG_CTC	6,241	-0,663	-0,774	-1,517	3,63E-04	3,09E-05	1,23E-15	5,30E-02	1,58E-02	2,52E-12
	3	GCC_GGC	5,011	-0,545	-0,758	-1,315	1,43E-02	6,82E-04	6,23E-09	4,00E-01	1,86E-01	2,55E-06
	4	AGC_GCT	4,827	-0,692	-0,528	-1,260	2,85E-03	2,08E-02	6,86E-08	2,04E-01	8,26E-01	1,90E-05
	5	GAA_TTC	4,790	-1,345	-1,532	-1,250	2,08E-07	4,05E-09	6,54E-07	4,03E-04	8,30E-06	1,22E-04
	6	TGA_TCA	4,588	-0,435	-0,894	-1,146	9,18E-02	6,56E-04	1,14E-05	6,59E-01	1,86E-01	9,18E-04
	7	GTG_CAC	5,700	-0,747	-1,201	-1,122	1,38E-04	1,58E-09	9,67E-09	3,23E-02	6,47E-06	3,60E-06
	8	GGT_ACC	3,830	-0,735	-1,268	-1,116	6,20E-03	4,52E-06	2,86E-05	2,86E-01	3,09E-03	1,97E-03
	9	CGA_TCG	3,746	-0,517	-0,556	-1,108	6,06E-02	4,13E-02	5,70E-05	6,00E-01	9,91E-01	3,16E-03
	10	TCG_CGA	5,060	-0,524	-0,607	-1,005	1,03E-02	2,84E-03	8,66E-07	3,64E-01	4,01E-01	1,54E-04

Tab. 3: Top 10 constructs of each Tet-Twister sub-library, ranked by their fold change at 50 µM Tet (here, no further cutoffs were applied).

Supplementary Table 4

Gua-HDV ribozyme library and hit validation

Name	Sequence	Comment
Gua-HDV ribozyme library fwd	tttctaccgggcaccgtaaatgtccgactaNNNgcgaatgggacgcacaaatct	Insertion of guanine xpt aptamer into HDV ribozyme with randomized communication module
Gua-HDV ribozyme library rev	cttgcgtgccatatccacgcgattataNNNttgcccagccggcg	
HDV act fwd	gaggggaccgtcccctcggtaatggcgaatgggacgcacaaatctctctagcaaacaaacaaagcctggtgaaattgttat	Insertion of HDV ribozyme
HDV act rev	ggaatgttgcccagccggcgccagcgaggaggctgggaccatgccggcctttgtttgtttgggactgggaatacg	
HDV inact fwd	tgaatgggacgcacaaatctctctag	Inactivation of HDV ribozyme
HDV inact rev	ccattaccgaggggacggt	
GuaM8HDV fwd	tttctaccgggcaccgtaaatgtccgactaGTAgcgaatgggacgcacaaatct	Insertion of guanine xpt aptamer into HDV ribozyme with GuaM8HDV communication module
GuaM8HDV rev	cttgcgtgccatatccacgcgattataGCAttgcccagccggcg	
GuaB1HDV fwd	tttctaccgggcaccgtaaatgtccgactaCTAgcgaatgggacgcacaaatct	Insertion of guanine xpt aptamer into HDV ribozyme with GuaB1HDV communication module
GuaB1HDV rev	cttgcgtgccatatccacgcgattataCCAttgcccagccggcg	
GuaB2HDV fwd	tttctaccgggcaccgtaaatgtccgactaTTAgcgaatgggacgcacaaatct	Insertion of guanine xpt aptamer into HDV ribozyme with GuaB2HDV communication module
GuaB2HDV rev	cttgcgtgccatatccacgcgattataTCAttgcccagccggcg	
GuaB3HDV fwd	tttctaccgggcaccgtaaatgtccgactaCTGgcgaatgggacgcacaaatct	Insertion of guanine xpt aptamer into HDV ribozyme with GuaB3HDV communication module
GuaB3HDV rev	cttgcgtgccatatccacgcgattataCCGttgcccagccggcg	
GuaB4HDV fwd	tttctaccgggcaccgtaaatgtccgactaTCGgcgaatgggacgcacaaatct	Insertion of guanine xpt aptamer into HDV ribozyme with GuaB4HDV communication module
GuaB4HDV rev	cttgcgtgccatatccacgcgattataCCGttgcccagccggcg	
GuaM12HDV fwd	tttctaccgggcaccgtaaatgtccgactaATAgcgaatgggacgcacaaatct	Insertion of guanine xpt aptamer into HDV ribozyme with GuaM12HDV communication module
GuaM12HDV rev	cttgcgtgccatatccacgcgattataACAttgcccagccggcg	
GuaB5HDV fwd	tttctaccgggcaccgtaaatgtccgactaACGgcgaatgggacgcacaaatct	Insertion of guanine xpt aptamer into HDV ribozyme with GuaB5HDV communication module
GuaB5HDV rev	cttgcgtgccatatccacgcgattataACGttgcccagccggcg	
GuaB6HDV fwd	tttctaccgggcaccgtaaatgtccgactaGCGgcgaatgggacgcacaaatct	Insertion of guanine xpt aptamer into HDV ribozyme with GuaB6HDV communication module
GuaB6HDV rev	cttgcgtgccatatccacgcgattataGCGttgcccagccggcg	
GuaB7HDV fwd	tttctaccgggcaccgtaaatgtccgactaGCGgcgaatgggacgcacaaatct	Insertion of guanine xpt aptamer into HDV ribozyme with GuaB7HDV communication module
GuaB7HDV rev	cttgcgtgccatatccacgcgattataACGttgcccagccggcg	
GuaB8HDV fwd	tttctaccgggcaccgtaaatgtccgactaTTGgcgaatgggacgcacaaatct	Insertion of guanine xpt aptamer into HDV ribozyme with GuaB8HDV communication module
GuaB8HDV rev	cttgcgtgccatatccacgcgattataTTGttgcccagccggcg	
GuaB9HDV fwd	tttctaccgggcaccgtaaatgtccgactaGTGgcgaatgggacgcacaaatct	Insertion of guanine xpt aptamer into HDV ribozyme with GuaB9HDV communication module
GuaB9HDV rev	cttgcgtgccatatccacgcgattataGCGttgcccagccggcg	
GuaB10HDV fwd	tttctaccgggcaccgtaaatgtccgactaGTGgcgaatgggacgcacaaatct	Insertion of guanine xpt aptamer into HDV ribozyme with GuaB10HDV communication module
GuaB10HDV rev	cttgcgtgccatatccacgcgattataGTGttgcccagccggcg	
Ampl fwd	agataaagcttggtacccattgcagcgtatt	Insertion of the constructs into CMV-eGFP pAAV expression plasmid backbone using HindIII and BglII restriction sites
Ampl rev	agataagatctagcggataacaatttcaccaggc	

Gua-HHR ribozyme library and hit validation

Name	Sequence	Comment
Gua-HHR ribozyme library fwd	tttctaccgggcaccgtaaatgtccgactaNNtacatccagctgatgagtcccaaatagg	Insertion of guanine xpt aptamer into HHR ribozyme with randomized communication module
Gua-HHR ribozyme library rev	cttgcgtgccatatccacgcgattataNNNNNgaatccaggacgcgcctttg	
Hh act fwd	cagctgatgagtcccaaataggacgaaacgcgctcaaacaaacaaagcctggtgaaattgttat	Insertion of HHR ribozyme
Hh act rev	gatgtaccgtggaatccaggacgcgcctttgtttgtttgggactgggaatacg	
Hh inact fwd	gacgcgctcaaacaaacaaa	Inactivation of HHR ribozyme
Hh inact rev	tcgtcctatttgggactc	
GuaB1HHR fwd	tttctaccgggcaccgtaaatgtccgactaTGtacatccagctgatgagtcccaaatagg	Insertion of guanine xpt aptamer into HHR ribozyme with GuaB1HHR communication module
GuaB1HHR rev	cttgcgtgccatatccacgcgattataAGTAGgaatccaggacgcgcctttg	
GuaB2HHR fwd	tttctaccgggcaccgtaaatgtccgactaCGtacatccagctgatgagtcccaaatagg	Insertion of guanine xpt aptamer into HHR ribozyme with GuaB2HHR communication module
GuaB2HHR rev	cttgcgtgccatatccacgcgattataGGTAGgaatccaggacgcgcctttg	
GuaB3HHR fwd	tttctaccgggcaccgtaaatgtccgactaCTtacatccagctgatgagtcccaaatagg	Insertion of guanine xpt aptamer into HHR ribozyme with GuaB3HHR communication module
GuaB3HHR rev	cttgcgtgccatatccacgcgattataGATACgaatccaggacgcgcctttg	
GuaB4HHR fwd	tttctaccgggcaccgtaaatgtccgactaAGtacatccagctgatgagtcccaaatagg	Insertion of guanine xpt aptamer into HHR ribozyme with GuaB4HHR communication module
GuaB4HHR rev	cttgcgtgccatatccacgcgattataACTACgaatccaggacgcgcctttg	
GuaB5HHR fwd	tttctaccgggcaccgtaaatgtccgactaGTtacatccagctgatgagtcccaaatagg	Insertion of guanine xpt aptamer into HHR ribozyme with GuaB5HHR communication module
GuaB5HHR rev	cttgcgtgccatatccacgcgattataGCTAGgaatccaggacgcgcctttg	
GuaB6HHR fwd	tttctaccgggcaccgtaaatgtccgactaGGtacatccagctgatgagtcccaaatagg	Insertion of guanine xpt aptamer into HHR ribozyme with GuaB6HHR communication module
GuaB6HHR rev	cttgcgtgccatatccacgcgattataAGTGCgaatccaggacgcgcctttg	
GuaB7HHR fwd	tttctaccgggcaccgtaaatgtccgactaTGtacatccagctgatgagtcccaaatagg	Insertion of guanine xpt aptamer into HHR ribozyme with GuaB7HHR communication module
GuaB7HHR rev	cttgcgtgccatatccacgcgattataAGTACgaatccaggacgcgcctttg	
Ampl fwd	agataaagcttggtacccattgcagcgtatt	Insertion of the constructs into CMV-eGFP pAAV expression plasmid backbone using HindIII and BglII restriction sites
Ampl rev	agataagatctagcggataacaatttcaccaggc	

TetTwister Hit validation

Name	Sequence	Comment
Tw act fwd	agatactcgagcaaacaaactcctttaaagcggttacaag	Amplification of Twister ribozyme and insertion into psiCHECK-2 plasmid using XhoI and NotI restriction sites
Tw act rev	agatagcggccgcttttctttttctcctttaaagctgcattaatg	
Tw inact fwd	agatactcgagcaaacaaactcctttaaagcgtgtacaag	Amplification of inactive Twister ribozyme and insertion into psiCHECK-2 plasmid using XhoI and NotI restriction sites
Tw inact rev	agatagcggccgcttttctttttctcctttaaagctgcattaatg	
T1 fwd	aggctcgaaagagcctaaaacatacCGGttaatgcagctttaaaggagaaaaagaaaagc	Insertion of tetracycline aptamer into Twister ribozyme with T1 communication module
T1 rev	aggtggtcgtattcttcacctctcCGttactctgctatttttgcgggcttg	
T2 fwd	aggctcgaaagagcctaaaacatacCCttaatgcagctttaaaggagaaaaagaaaagc	Insertion of tetracycline aptamer into Twister ribozyme with T2 communication module
T2 rev	aggtggtcgtattcttcacctctcCCGttactctgctatttttgcgggcttg	
T3 fwd	aggctcgaaagagcctaaaacatacCGTttaatgcagctttaaaggagaaaaagaaaagc	Insertion of tetracycline aptamer into Twister ribozyme with T3 communication module
T3 rev	aggtggtcgtattcttcacctctcCGGttactctgctatttttgcgggcttg	
T4 fwd	aggctcgaaagagcctaaaacatacCTTttaatgcagctttaaaggagaaaaagaaaagc	Insertion of tetracycline aptamer into Twister ribozyme with T4 communication module
T4 rev	aggtggtcgtattcttcacctctcCTCttactctgctatttttgcgggcttg	
T5 fwd	aggctcgaaagagcctaaaacatacCGAgttaatgcagctttaaaggagaaaaagaaaagc	Insertion of tetracycline aptamer into Twister ribozyme with T5 communication module
T5 rev	aggtggtcgtattcttcacctctcCGgttactctgctatttttgcgggcttg	
T6 fwd	aggctcgaaagagcctaaaacatacCGGgttaatgcagctttaaaggagaaaaagaaaagc	Insertion of tetracycline aptamer into Twister ribozyme with T6 communication module
T6 rev	aggtggtcgtattcttcacctctcCGgttactctgctatttttgcgggcttg	
T7 fwd	aggctcgaaagagcctaaaacatacCTgttaatgcagctttaaaggagaaaaagaaaagc	Insertion of tetracycline aptamer into Twister ribozyme with T7 communication module
T7 rev	aggtggtcgtattcttcacctctcCTGgttactctgctatttttgcgggcttg	
T8 fwd	aggctcgaaagagcctaaaacatacCGgttaatgcagctttaaaggagaaaaagaaaagc	Insertion of tetracycline aptamer into Twister ribozyme with T8 communication module
T8 rev	aggtggtcgtattcttcacctctcCGGgttactctgctatttttgcgggcttg	
T9 fwd	aggctcgaaagagcctaaaacatacCAGgttaatgcagctttaaaggagaaaaagaaaagc	Insertion of tetracycline aptamer into Twister ribozyme with T9 communication module
T9 rev	aggtggtcgtattcttcacctctcCGGgttactctgctatttttgcgggcttg	
T10 fwd	aggctcgaaagagcctaaaacatacGGCgttaatgcagctttaaaggagaaaaagaaaagc	Insertion of tetracycline aptamer into Twister ribozyme with T10 communication module
T10 rev	aggtggtcgtattcttcacctctcGCCgttactctgctatttttgcgggcttg	
Neg. control #500 fwd	aggctcgaaagagcctaaaacatacTCCgttaatgcagctttaaaggagaaaaagaaaagc	Insertion of tetracycline aptamer into Twister ribozyme with communication module of neg. control #500
Neg. control #500 rev	aggtggtcgtattcttcacctctcCTGgttactctgctatttttgcgggcttg	
Neg. control #1000 fwd	aggctcgaaagagcctaaaacatacGACgttaatgcagctttaaaggagaaaaagaaaagc	Insertion of tetracycline aptamer into Twister ribozyme with communication module of neg. control #1000
Neg. control #1000 rev	aggtggtcgtattcttcacctctcACAgttactctgctatttttgcgggcttg	
Neg. control #2000 fwd	aggctcgaaagagcctaaaacatacGCTgttaatgcagctttaaaggagaaaaagaaaagc	Insertion of tetracycline aptamer into Twister ribozyme with communication module of neg. control #2000
Neg. control #2000 rev	aggtggtcgtattcttcacctctcAGGgttactctgctatttttgcgggcttg	
Neg. control #3000 fwd	aggctcgaaagagcctaaaacatacCCCgttaatgcagctttaaaggagaaaaagaaaagc	Insertion of tetracycline aptamer into Twister ribozyme with communication module of neg. control #3000
Neg. control #3000 rev	aggtggtcgtattcttcacctctcAGGgttactctgctatttttgcgggcttg	

Tab. 4: Cloning primers used in this study.

Supplementary Table 4 - continued

Gua-U1 library and hit validation

Name	Sequence	Comment
Gua-U1 library fwd	tttctaccgggcaccgtaaatgtccgactaNNNacttacctgcaaacaaacaaagcctggtgaaattgttat	Insertion of guanine xpt aptamer onto U1 snRNP binding site with randomized communication module
Gua-U1 library rev	cttgcgtgccatatccacgcgattataNNNacttacctgtttgtttgtttgggactgggaatacg	
U1 bs fwd	caaacaaacaaagcctggtgaaattgttat	Insertion of U1 snRNP binding site
U1 bs rev	acttacctgtttgtttgtttgggactgggaatacg	
GuaU1B1 fwd	tttctaccgggcaccgtaaatgtccgactaGCGacttacctgcaaacaaacaaagcctggtgaaattgttat	Insertion of guanine xpt aptamer onto U1 snRNP binding site with GuaU1B1 communication module
GuaU1B1 rev	cttgcgtgccatatccacgcgattataGCCacttacctgtttgtttgtttgggactgggaatacg	
GuaU1B2 fwd	tttctaccgggcaccgtaaatgtccgactaGGCacttacctgcaaacaaacaaagcctggtgaaattgttat	Insertion of guanine xpt aptamer onto U1 snRNP binding site with GuaU1B2 communication module
GuaU1B2 rev	cttgcgtgccatatccacgcgattataGTCacttacctgtttgtttgtttgggactgggaatacg	
GuaU1B3 fwd	tttctaccgggcaccgtaaatgtccgactaAGTacttacctgcaaacaaacaaagcctggtgaaattgttat	Insertion of guanine xpt aptamer onto U1 snRNP binding site with GuaU1B3 communication module
GuaU1B3 rev	cttgcgtgccatatccacgcgattataAGCacttacctgtttgtttgtttgggactgggaatacg	
GuaU1B4 fwd	tttctaccgggcaccgtaaatgtccgactaAGGacttacctgcaaacaaacaaagcctggtgaaattgttat	Insertion of guanine xpt aptamer onto U1 snRNP binding site with GuaU1B4 communication module
GuaU1B4 rev	cttgcgtgccatatccacgcgattataAGAacttacctgtttgtttgtttgggactgggaatacg	
GuaU1B5 fwd	tttctaccgggcaccgtaaatgtccgactaGTTacttacctgcaaacaaacaaagcctggtgaaattgttat	Insertion of guanine xpt aptamer onto U1 snRNP binding site with GuaU1B5 communication module
GuaU1B5 rev	cttgcgtgccatatccacgcgattataGTCacttacctgtttgtttgtttgggactgggaatacg	
GuaU1B6 fwd	tttctaccgggcaccgtaaatgtccgactaTGCacttacctgcaaacaaacaaagcctggtgaaattgttat	Insertion of guanine xpt aptamer onto U1 snRNP binding site with GuaU1B6 communication module
GuaU1B6 rev	cttgcgtgccatatccacgcgattataTCCacttacctgtttgtttgtttgggactgggaatacg	
GuaU1B7 fwd	tttctaccgggcaccgtaaatgtccgactaAGCacttacctgcaaacaaacaaagcctggtgaaattgttat	Insertion of guanine xpt aptamer onto U1 snRNP binding site with GuaU1B7 communication module
GuaU1B7 rev	cttgcgtgccatatccacgcgattataACCacttacctgtttgtttgtttgggactgggaatacg	
GuaU1B8 fwd	tttctaccgggcaccgtaaatgtccgactaACTacttacctgcaaacaaacaaagcctggtgaaattgttat	Insertion of guanine xpt aptamer onto U1 snRNP binding site with GuaU1B8 communication module
GuaU1B8 rev	cttgcgtgccatatccacgcgattataACCacttacctgtttgtttgtttgggactgggaatacg	
GuaU1B9 fwd	tttctaccgggcaccgtaaatgtccgactaATTacttacctgcaaacaaacaaagcctggtgaaattgttat	Insertion of guanine xpt aptamer onto U1 snRNP binding site with GuaU1B9 communication module
GuaU1B9 rev	cttgcgtgccatatccacgcgattataATCacttacctgtttgtttgtttgggactgggaatacg	
GuaU1B10 fwd	tttctaccgggcaccgtaaatgtccgactaTACacttacctgcaaacaaacaaagcctggtgaaattgttat	Insertion of guanine xpt aptamer onto U1 snRNP binding site with GuaU1B10 communication module
GuaU1B10 rev	cttgcgtgccatatccacgcgattataTGCacttacctgtttgtttgtttgggactgggaatacg	
Ampl fwd	agataaagcttggtacccattgcagcgtatt	Insertion of the constructs into CMV-eGFP pAAV expression plasmid backbone using HindIII and BglII restriction sites
Ampl rev	agataagatctagcggataacaatttcaccaggc	

Supplementary Table 5

TwisterTet_3N2N	AGTAA([ACGT]{3})GAGAGG.{37}ACATAC([ACGT]{2})TTAAT
TwisterTet_2N3N	AGTAA([ACGT]{2})GAGAGG.{37}ACATAC([ACGT]{3})TTAAT
TwisterTet_3N3N	AGTAA([ACGT]{3})GAGAGG.{37}ACATAC([ACGT]{3})TTAAT
TwisterTet_CG_3N2N	AGTAAC([ACGT]{3})GAGAGG.{37}ACATAC([ACGT]{2})GTTAAT
TwisterTet_CG_2N3N	AGTAAC([ACGT]{2})GAGAGG.{37}ACATAC([ACGT]{3})GTTAAT
TwisterTet_CG_3N3N	AGTAAC([ACGT]{3})GAGAGG.{37}ACATAC([ACGT]{3})GTTAAT
TwisterTet_noCG_3N2N	AGTAA([ACGT]{3})AGAGG.{37}ACATA([ACGT]{2})TTAAT
TwisterTet_noCG_2N3N	AGTAA([ACGT]{2})AGAGG.{37}ACATA([ACGT]{3})TTAAT
TwisterTet_noCG_3N3N	AGTAA([ACGT]{3})AGAGG.{37}ACATA([ACGT]{3})TTAAT
Spikein	TTAGTAACAG

Tab. 5: Regular expressions used in the variant extraction.
